# Immunoglobulin E and G Levels in Predicting Minimal Change Disease before Renal Biopsy

**DOI:** 10.1155/2018/3480309

**Published:** 2018-11-11

**Authors:** Ching-Chung Hsiao, Kun-Hua Tu, Chun-Yih Hsieh, Cheng-Chia Lee, Chih-Hsiang Chang, Pei-Chun Fan, Ya-Chung Tian, Ji-Tseng Fang

**Affiliations:** ^1^Kidney Research Center, Department of Nephrology, Chang Gung Memorial Hospital, Taoyuan, Taiwan; ^2^College of Medicine, Chang Gung University, Taoyuan, Taiwan; ^3^Graduate Institute of Clinical Medical Sciences, Chang Gung University, Taoyuan, Taiwan

## Abstract

**Purpose:**

The diagnosis of minimal change disease in adults relies mainly on renal biopsy, but this procedure is not without complications. Despite the advancements in technique of percutaneous renal biopsy, biopsy-related complications still occur. Bleeding is one of the major complications, which may lead to hemodynamic instability and, sometimes, even death. Thus, we developed a model to predict MCD for high-risk patients unsuitable for renal biopsy.

**Methods:**

We enrolled 142 patients with nephrotic syndrome who received renal biopsy between October 2007 and April 2011 at one tertiary medical center in this study. Demographic, clinical, and prebiopsy laboratory variables were retrospectively recorded and analyzed.

**Results:**

The overall prevalence of MCD was 26.8%. Age, hemoglobin levels, 24-hour urine protein, immunoglobulin (Ig) G, and IgE differed significantly between the MCD and non-MCD groups. Logistic regression analysis showed a significant increase in the risk of developing MCD as the number of Ig risk factors, namely, IgG < 450 mg/dl and IgE > 110 mg/dl, increased. Having both risk factors significantly increased the chances of receiving a diagnosis of MCD (by 31.84-fold, P =.007) compared with having neither. Combining the aforementioned clinical model and the 2 Ig risk factors was the best in predicting the diagnosis of MCD, with the area under a receiver-operating characteristic curve of 0.91.

**Conclusions:**

Combining clinical model and this 2 Ig risk factors provides physicians simple and valuable clinical markers to diagnose MCD.

## 1. Introduction

Minimal change disease (MCD) is not only the most common disease underlying childhood nephrotic syndrome, but also a major cause of nephrotic syndrome in adults. Approximately 10–15% of adult-onset nephrotic syndrome results from MCD. Diagnosis of nephrotic syndrome in children is usually based on clinical presentation, and renal biopsy is not routinely required. The International Study of Kidney Disease in Children series demonstrated that MCD accounts for 70–90% of nephrotic syndrome in children, and that 93% of children with MCD and 25–50% of children with focal segmental glomerulosclerosis (FSGS) or mesangial proliferative glomerulonephritis (MPGN) also respond to corticosteroids [1]. Thus, corticosteroids should be empirically given for nephrotic syndrome in children. Renal biopsy is only indicated for corticosteroid resistance, late treatment failure, and suspicion of different pathologic diagnosis [2].

Unlike in children, MCD accounts for a lower percentage of nephrotic syndrome in adults. In addition, adults with MCD have different clinical features, have a high incidence of concomitant acute kidney injury, and do not always respond to corticosteroid [3]. Therefore, renal biopsy is a prerequisite for adults with nephrotic syndrome, and the diagnosis of MCD often relies on this invasive procedure. Although there have been advances in imaging technology and interventional tools of percutaneous renal biopsy, postbiopsy complications can still occur. Bleeding is one of the major primary complications. Rarely, renal biopsy complicates with major hemorrhage, necessitating surgical intervention such as nephrectomy or even leading to death. Careful evaluation of risks and benefits must be done before the procedure. In patients with bleeding risk or other contraindications to renal biopsy, physicians must initiate treatment based merely on clinical presentation and personal experience.

The major aim of this study is to identify prebiopsy serologic markers that can predict the diagnosis of MCD in adults with nephrotic syndrome. The development of the model could provide physicians one more evaluation tool, especially when renal biopsy is contraindicated.

## 2. Methods

### 2.1. Patient Information and Data Collection

The Institutional Review Board of Chang Gung Memorial Hospital approved the study and waived the need for informed consent because there was no breach of privacy and the study did not interfere with clinical decisions related to patient care (approval No. 201600235B0). Patients with nephrotic range proteinuria or nephrotic syndrome who received renal biopsy at Chang Gung Memorial Hospital, a university-affiliated tertiary referral center in Taiwan, during October 2007–April 2011 were enrolled. Nephrotic range proteinuria was defined as a daily urinary protein loss more than 3gm. The nephrotic syndrome was defined as the combination of nephrotic range proteinuria with a low albumin levels (< 2.5 g/dl) and edema. Patients with any of the following were excluded: estimated glomerular filtration rate <30ml/min (modification of diet in renal disease formula); clinical or pathological features indicating a secondary cause such as autoimmune diseases, chronic infections especially hepatitis B or C, cancer, and exposure to causative drugs. Finally, 142 patients were included in this study. Demographic characteristics, clinical and laboratory variables before renal biopsy, indications of renal biopsy, and histopathology reports were retrospectively collected by review of medical charts; and all data were anonymized.

### 2.2. Definition

The sample for histopathological diagnosis was taken using sonography-guided percutaneous renal biopsy performed before initiating corticosteroid or other immunosuppressive therapy. The specimens were processed for light microscopy, immunofluorescence, and electron microscopy following standard procedures and were reviewed by experienced renal pathologists.

The diagnosis of MCD was made based on normal-appearing glomeruli on light microscopy; negative or nonspecific staining of immunoglobulin and complement on immunofluorescence; and diffuse foot process effacement of podocytes and absence of electron-dense deposits on electron microscopy.

### 2.3. Statistical Analysis

Continuous variables were summarized using means ± standard deviation. The primary analysis was to compare MCD patients with non-MCD patients. All variables were tested for normal distribution by using the Kolmogorov–Smirnov test. The Student's t-test was used to compare the means of continuous variables and normally distributed data; otherwise, the Mann–Whitney U test was used. Categorical data were tested using the chi-square test or Fisher exact test. Discrimination was assessed by using the area under a receiver-operating characteristic curve (AUROC). Two AUROCs were compared by a nonparametric approach. The AUROC analysis was also performed to calculate cutoff values, sensitivity, and specificity. Finally, cutoff points were calculated by acquiring the best Youden index (sensitivity + specificity − 1). Multivariate logistic regression analysis was performed to evaluate factors associated with MCD. All statistical tests were 2-tailed, with the level of significance set at* P* <.05. Data were analyzed using SPSS 19.0 for Windows (SPSS, Inc., Chicago, IL, USA).

## 3. Results

### 3.1. Patient Characteristics

The study enrolled 142 patients with a mean age (at biopsy) of 42.4 ± 17.0 years; 57.7 % of patients were male, and 38 (26.8%) were diagnosed with MCD.  Table 1 lists the comparison of baseline characteristics and laboratory parameters between the 2 groups. Compared with the non-MCD group, patients in the MCD group were younger, had a male preponderance, and exhibited higher values of hemoglobin and 24-hour urinary protein. With regard to serology markers, patients in the MCD group had a significantly lower serum IgG level than those in the non-MCD group (*P *=.001). Serum IgE level in the MCD group was significantly higher than that of the non-MCD group (*P* <.001). We did not observe a significant difference in serum IgA and IgM levels between the 2 groups.

### 3.2. Performance of IgG and IgE in the Diagnosis of MCD

Table 2 lists the results of the AUROC analysis and optimal cutoff values with corresponding sensitivity and specificity. Among the 3 clinical well-established factors, hemoglobin level displayed the highest AUROC of 0.847 (95% confidence interval [CI] 0.761–0.932,* P*<0.001), followed by age (AUROC 0.807,* P *<.001) and 24-hour urinary protein (AUROC 0.703,* P *=.002). IgG and IgE exhibited similar and satisfactory discriminating power in predicting the diagnosis of MCD, with AUROCs of 0.748 and 0.781, respectively. A cutoff IgE value of 110 mg/dl, as determined by the Youden index, exhibited the best sensitivity of 92% and specificity of 56%. For IgG, an optimal cutoff value of 450 mg/dl yielded sensitivity of 70% and specificity of 76%.

### 3.3. Logistic Regression Analysis of Factors Associated with the Diagnosis of MCD

To determine whether IgG and IgE measurement could help physicians to predict the diagnosis of MCD, univariate and multivariate logistic regression analyses were conducted (Table 3). When the parameters of IgG < 450 mg/dl and IgE > 110 mg/dl were alternatively added to the multivariate model, the HRs for the diagnosis of MCD were 4.29 (95% CI, 1.07–17.20;* P *=.04) and 9.14 (95% CI, 1.74–47.89,* P *=.009), respectively.

**Figure 1:**
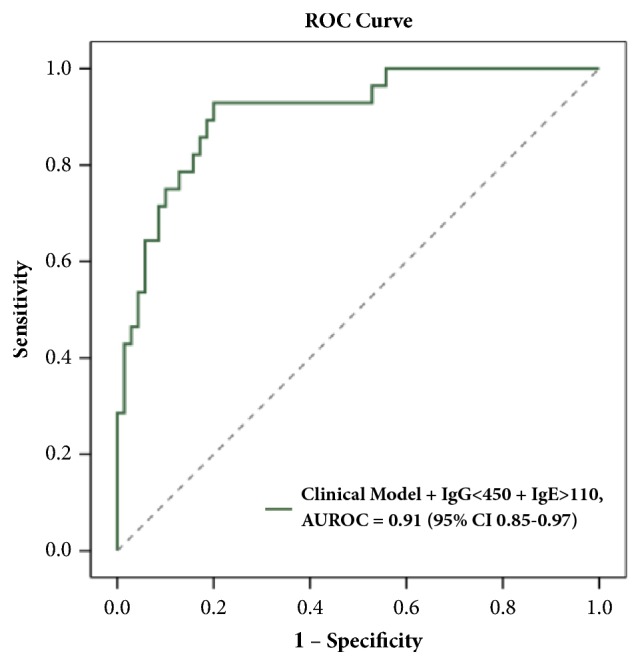
Receiver-operating characteristic curve of combination of the clinical model with IgG+IgE in predicting the diagnosis of MCD.

We further divided the patients into 3 groups on the basis of whether none, one, or both of the risk factors, namely, IgG < 450 mg/dl and IgE > 110 mg/dl, were present. With the number of risk factors increased, there were stepwise increases in the HR of the diagnosis of MCD after adjustment for all significant factors. Having both risk factors significantly increased the HRs of a patient to have MCD by 31.84-fold (*P *=.007) compared with having neither of them. Overall, our study demonstrated that a combination of the existing clinical model with these 2 Ig risk factors was excellent in predicting the diagnosis of MCD, with the AUROC of 0.91 (95% CI 0.85–0.97,* P *<.001; Figure 1).

## 4. Discussion

MCD is characterized histologically by the normal-appearing glomeruli on light microscopy and diffuse foot process effacement and absence of electron-dense deposits on electron microscopy. MCD is believed to be a type of podocytopathy. Loss of negatively charged glycocalyx of podocytes results in not only urinary leakage of the negatively charged protein, mainly albumin, but also extensive foot process effacement [4]. The underlying mechanism, however, remains unclear, and many hypotheses have been recently proposed. Some investigators suggested the existence of circulating permeability factors, including hemopexin [5] and angiopoietin-like-4 (ANGPTL4) [6], which could interact with the glycocalyx and cause the loss of charge-selective barrier.

Our finding for the performance of increased serum IgE levels in predicting MCD is consistent with some earlier studies [7–10]. In recent years, it has become evident that the underlying immune dysfunction in these patients predisposes them to developing both nephrotic syndrome and increased serum IgE levels [11]. Production of IgE by B cells requires 2 signals: the first is driven by 2 cytokines IL-4 and IL-13 released by Th2 cells, and the second is initiated by the interaction of the B-cell surface antigen CD40 with CD40 ligand expressed on activated T cells [12]. Critically, IL-13 was considered as a potential mediator of MCD in several studies [13, 14]. Intracellular expression of IL-13 in T cells is directly associated with serum IgE levels [15]. Several studies also revealed increased serum IL-13 levels in patients with MCD [16]. Thus, it is conceivable that increased serum IgE level is a downstream product of IL-13 activation and indirectly associated with MCD. Future studies are warranted to investigate this possibility.

Although there are acceptable hypotheses for the mechanism of MCD, none can fully explain the entire clinical and histologic picture. Nevertheless, the aforementioned implicated molecules are clinically applied as predictors of the diagnosis of MCD. Recent studies suggest that IL-13 can induce upregulation of CD80 on podocyte, leading to podocyte effacement and proteinuria [17]. Chen Ling et al. proposed the ratio of urinary CD80 and creatinine, with a cutoff value of 328.98 (ng/g creatinine), as a predictor of the diagnosis of MCD, with a sensitivity of 81.1% and specificity of 94.4% [18]. The AUROC for the urinary CD80 to diagnose MCD was 0.925 (95% confidence interval: 0.873–0.978). Whether serum IL-13 or IgE levels increase in proportion to the urinary CD80 levels in our study is not clear. Additional studies are needed to investigate their relationship.

Studies have revealed decreased IgG and increased IgM levels during the relapse of steroid-sensitive nephrotic syndrome [19–21]. Disproportional depression of IgG subclasses, especially IgG1 and IgG2, causes the decrease of serum total IgG level during relapses [22, 23]. Decreased IgG level may result from urinary loss of IgG or impaired class switch from IgM to IgG [22]. Our study found significantly decreased IgG level in patients with MCD, but IgM level was not increased.

Few clinical risk prediction models for MCD have been developed. In the present study, age, 24-hour urinary protein, and elevated hemoglobin levels are good indicators for MCD even under adjusted models. A patient younger than 34.5 years old with proteinuria >10.9 g and a hemoglobin level >13.7 g/dL is more likely to have MCD. Young age and heavy proteinuria are typical clinical features of MCD. Regarding hematologic parameters, hemoconcentration leading to increased hemoglobin levels is frequently observed in patients with MCD. As a single predictor, hemoglobin levels > 13.7 g/dl adopted best discriminative power in predicting MCD. Notably, Qin et al. reported that a cohort of IgA nephropathy patients who exhibited MCD-like pathological changes had a higher level of hemoglobin when compared with those who did not exhibit MCD-like lesions [24].

Furthermore, combining the clinical model and these 2 Ig risk factors (IgG<450mg/dl and IgE>110mg/dl) exhibited similar and excellent discrimination in predicting the diagnosis of MCD compared with urinary CD80 from prior report [18]. The measurement of urinary CD80 is more expensive and time consuming than that of serum Ig; also, it is not readily available in general hospitals. Thus, our new proposed model provides the physician simple and valuable clinical markers with which MCD is diagnosed. Patients with nephrotic syndrome with absolute or relative contraindications to renal biopsy may benefit from this prediction model.

Despite the favorable results, our study has some crucial limitations. First, we employed a post hoc design in a single referral center; generalization of our results should be done with caution. Second, a biopsy was not routinely performed in each patient with nephrotic syndrome suspected with MCD, and exclusion of such patients might influence the cutoff. Further prospective study to verify this result might increase the accuracy. Finally, lack of follow-up data also limited our attempt to correlate these predictors with disease activity, and repeated measurements might further improve the discrimination.

In conclusion, we found that age, hemoglobin, and 24-hour urinary protein are significant predictors of MCD before renal biopsy. Combining this with the 2 Ig risk factors (IgG < 450mg/dl and IgE > 110mg/dl) best predicted the diagnosis of MCD. This set of parameters provides the physician with simple and valuable clinical markers to diagnose MCD. Further studies are warranted to examine the role of Igs as diagnostic, pathogenic, and prognostic markers in MCD.

## Figures and Tables

**Table 1:** Baseline characteristics of patients with or without minimal change disease (MCD).

Characteristics	All (n = 142)	MCD (n = 38)	non-MCD (n = 104)	*p* value

Age (years)	42.4 ± 17.0	29.9 ± 13.3	46.8 ± 15.9	<0.001
Men [n (%)]	82 (57.7%)	28 (73.7%)	54 (51.9%)	0.022
WBC (x10^3^/mm^3^)	7.9 ± 3.0	7.6 ± 2.6	8.0 ± 3.2	0.562
Hemoglobin (g/dl)	12.9 ± 2.4	14.7 ± 2.2	12.2 ± 2.1	<0.001
Platelet count (x10^3^/mm^3^)	264 ± 75	276 ± 65	260 ± 78	0.258
Blood urea nitrogen (mg/dl)	19.6 ± 11.7	20.8 ± 15.3	19.2 ± 10.1	0.486
Creatinine (mg/dl)	0.93 ± 0.40	0.88 ± 0.36	0.95 ± 0.42	0.313
Total protein (g/dl)	5.1 ± 1.1	4.8 ± 1.3	5.2 ± 1.0	0.108
Serum sodium (meq/L)	140.2 ± 3.5	139.2 ± 3.8	140.5 ± 3.4	0.071
Serum Potassium (meq/L)	4.0 ± 0.5	4.1 ± 0.5	3.9 ± 0.5	0.109
Proteinuria (g/day)	8.7 ± 7.3	13.2 ± 8.5	7.0 ± 5.9	<0.001
24 hours urine output (ml)	1951 ± 972	1950 ± 1130	1951 ± 915	0.998
Microscopic hematuria [n (%)]	3 (2.1%)	0 (0.0%)	3 (2.9%)	0.564
***Serology markers***				
Immunoglobulin G (mg/dl)	681.2 ± 381.5	500.7 ± 411.1	757.7 ± 347.2	0.001
Immunoglobulin M (mg/dl)	123.7 ± 68.8	127.0 ± 48.6	122.4 ± 75.2	0.751
Immunoglobulin A (mg/dl)	287.6 ± 113.8	269.1 ± 102.4	294.7 ± 117.6	0.253
Immunoglobulin E (mg/dl)	187 (47-639)	597 (146-1940)	108 (29-367)	<0.001

Data are presented as mean ± SD, n (%), or median (interquartile range) unless otherwise specified.

The difference between 2 groups was determined by t-test or chi-square test for normally distributed variables and by Mann–Whitney *U* test for non-normally distributed variables.

**Table 2:** Discrimination, sensitivity, and specificity of factors in predicting minimal change disease.

	Discrimination			Optimal Cutoff	Youden Index	Sensitivity (%)	Specificity (%)
	AUROC ± SE	95% CI	*p*				
Age	0.807 ± 0.047	0.715-0.899	<0.001	34.5	0.53	75	78
Hemoglobin	0.847 ± 0.043	0.761-0.932	<0.001	13.7	0.61	78	83
Proteinuria	0.703 ± 0.065	0.576-0.830	0.002	10.9	0.44	63	81
IgG	0.748 ± 0.058	0.635-0.861	<0.001	450	0.48	70	76
IgE	0.781 ± 0.048	0.687-0.875	<0.001	110	0.48	92	56

**Table 3:** Univariate and multivariate logistic regression analyses of factors associated with minimal change disease.

Potential factors	Univariate		Multivariate					
			Model 1		Model 2		Model 3	
	HR (95% CI)	*P* value	HR (95% CI)	*P* value	HR (95% CI)	*P* value	HR (95% CI)	*P* value
Age, per year	0.92 (0.89-0.95)	<0.001	0.94 (0.90-0.98)	0.004	0.94 (0.91-0.98)	0.005	0.95 (0.91-0.99)	0.011
Sex (men)	2.59 (1.14-5.88)	0.02	0.58 (0.13-2.59)	0.48	0.79 (0.17-3.60)	0.76	0.51 (0.10-2.63)	0.47
Hemoglobin, per g/dl	1.81 (1.43-2.30)	<0.001	1.68 (1.10-2.57)	0.016	1.57 (1.06-2.33)	0.024	1.61 (1.05-2.47)	0.029
Proteinuria, per g/day	1.13 (1.06-1.20)	<0.001	1.04 (0.95-1.13)	0.40	1.05 (0.97-1.14)	0.22	1.00 (0.92-1.09)	0.95
IgG < 450 mg/dl	8.12 (3.29-20.1)	<0.001	4.29 (1.07-17.20)	0.04	-	-		
IgE > 110 mg/dl	10.32 (2.90-36.7)	<0.001	-	-	9.14 (1.74-47.89)	0.009		
***Risk serology markers: IgG < 450 mg/dl, IgE > 110 mg/dl***								0.02
1 *versus* 0 risk markers							7.34 (0.76-70.71)	0.085
2 *versus* 0 risk markers							31.84 (2.55-397.3)	0.007

## Data Availability

Numerical data is available to interested readers upon request to the corresponding author of this article.

## Abbreviations

MCD: Minimal change disease
FSGS: Focal segmental glomerulosclerosis
Ig: Immunoglobulin.
